# Preparation of La_0.7_Ca_0.3−x_Sr_x_MnO_3_ Manganites by Four Synthesis Methods and Their Influence on the Magnetic Properties and Relative Cooling Power

**DOI:** 10.3390/ma12020309

**Published:** 2019-01-19

**Authors:** María E. Botello-Zubiate, María C. Grijalva-Castillo, Daniel Soto-Parra, Renee J. Sáenz-Hernández, Carlos R. Santillán-Rodríguez, José A. Matutes-Aquino

**Affiliations:** 1 Centro de Investigación en Materiales Avanzados, S.C., Miguel de Cervantes 120, Complejo Industrial Chihuahua, Chihuahua 31136, Mexico; eugenia.botello@cimav.edu.mx (M.E.B.-Z.); joselin.saenz@cimav.edu.mx (R.J.S.-H.); carlos.santillan@cimav.edu.mx (C.R.S.-R.); 2 CONACYT-Centro de Investigación en Materiales Avanzados, S.C., Miguel de Cervantes 120, Complejo Industrial Chihuahua, Chihuahua 31136, Mexico; cristina.grijalva@cimav.edu.mx; 3 Tecnológico Nacional de México/Instituto Tecnológico de Delicias, Paseo Tecnológico km. 3.5, Cd. Delicias, Chihuahua 33000, Mexico; enrique.soto.parra@gmail.com

**Keywords:** magnetic refrigeration, relative cooling power, spark plasma sintering

## Abstract

Manganites of the family La_0.7_Ca_0.3−x_Sr_x_MnO_3_ were fabricated by four preparation methods: (a) the microwave-assisted sol-gel Pechini method; (b) sol-gel Pechini chemical synthesis; (c) solid-state reaction with a planetary mill; and (d) solid-state reaction with an attritor mill, in order to study the effect of the preparation route used on its magnetocaloric and magnetic properties. In addition, the manganites manufactured by the Pechini sol-gel method were compacted using Spark Plasma Sintering (SPS) to determine how the consolidation process influences its magnetocaloric properties. The Curie temperatures of manganites prepared by the different methods were determined in ~295 K, with the exception of those prepared by a solid-state reaction with an attritor mill which was 301 K, so there is no correlation between the particle size and the Curie temperature. All samples gave a positive slope in the Arrot plots, which implies that the samples underwent a second order Ferromagnetic (FM)–Paramagnetic (PM) phase transition. Pechini sol-gel manganite presents higher values of Relative Cooling Power (RCP) than the solid-state reaction manganite, because its entropy change curves are smaller, but wider, associated to the particle size obtained by the preparation method. The SPS technique proved to be easier and faster in producing consolidated solids for applications in active magnetic regenerative refrigeration compared with other compaction methods.

## 1. Introduction

Magnetic refrigeration, based on the magnetocaloric effect, is a promising technology due to its greater cooling efficiency, the possibility of building more compact (even portable) devices, and because it is environmentally friendlier than the conventional cooling processes based on the compression and expansion cycles of gas [1]. In recent years, there has been an increasing amount of investigations related to magnetocaloric materials, such as rare-earth-based intermetallic compounds [2,3,4,5,6], perovskite-type oxides [7], rare-earth cuprates [8], and others.

Among them, La(Ca,Sr)MnO_3_ manganites are considered the most suitable materials to be used in magnetic refrigeration, owing to their physical properties, such as small magnetic and thermal hysteresis, a large magnetocaloric effect around the paramagnetic to ferromagnetic transition temperature (Curie temperature, T_C_), and broad working temperature ranges due to easy controlling of the Curie temperature by doping [9,10,11]. In addition, manganites have low preparation costs, chemical stability (no oxidation), and high electrical resistance [12,13]. 

The ability to tune the Curie temperature, as well as the ability to build a multiple-material regenerator in a practical way [14,15] makes the manganites important materials for use in magnetic refrigeration devices. Because of this, numerous investigations have been carried out on the effect of the preparation method in the magnetic and magnetocaloric properties of manganites [1,11,16,17,18,19,20]. 

La_0.7_Ca_0.3_MnO_3_ has a higher magnetocaloric effect (MCE) than La_0.7_Sr_0.3_MnO_3_, but a Curie temperature below room temperature. Doping Ca manganite with small amounts of Sr increases the Curie temperature to values closer to room temperature. This is important for application in magnetic refrigeration, but at the expenses of a lower MCE [21]. 

In this study, La(Ca,Sr)MnO manganites were fabricated by four different preparation methods: microwave-assisted sol-gel Pechini synthesis, sol-gel Pechini chemical synthesis, a solid-state reaction route with a planetary mill, and a solid-state reaction route with an attritor mill, each obtaining different morphologies, particle sizes, and chemical compositions which exert their influence on magnetocaloric properties.

## 2. Materials and Methods 

La, Ca, and Sr manganites with nominal composition La_0.7_Ca_0.3−x_Sr_x_MnO_3_ (x = 0.04, 0.06, and 0.07), were prepared by four different methods: sol-gel Pechini, microwave-assisted sol-gel Pechini, solid-state reaction route with planetary ball mill, and solid-state reaction route with attritor mill. The objective was to obtain a magnetocaloric material with a Curie temperature close to 295 K, suitable for applications in a magnetic regenerator for magnetic refrigeration. Therefore, slightly different compositions were prepared around the nominal composition La_0.7_Ca_0.25_Sr_0.05_MnO_3_. Finally, we selected, for each preparation method, a composition with a Curie temperature as close to 295 K as possible.

The preparation methods are described below, and the most important results obtained so far are presented.

**Sol-gel Pechini chemical synthesis.** Stoichiometric quantities of lanthanum nitrate (La(NO_3_)_3.6_H_2_O) 99.999%, calcium nitrate (Ca(NO_2_)_3.4_H_2_O) 99.0%, strontium nitrate (Sr(NO_2_)_3_) 99.0%, and manganese nitrate (Mn(NO_2_)_3.4_H_2_O) 97.0% were dissolved in distilled water with continuous magnetic stirring; then, citric acid and ethylene glycol were added, and the solution was maintained at 75 °C until the formation of a gel. The nitrates-to-citric-acid molar ratio was 1:1. The obtained gel was heated at a rate of 10 °C/min up to 250 °C, and maintained at this temperature for 14 h. After that, two thermal treatments, one at 550 °C for 3 h and another at 1000 °C for 10 h, were carried out. The obtained powders were pressed and sintered at 1200 °C for 6 h [22,23]. Also, powders obtained by this method were sintered in a Fuji Dr. Sinter Lab Jr. Spark Plasma Sintering (SPS) system, model 211Lx. Tablets of 28.5 mm were compacted using a current of 650 A, with a maximum pressure of 14.7 kN and a maximum temperature of 750 °C for 30 min. SPS allows for sample compaction with high density, low porosity, and almost without changes in microstructure size [24]. The sintering process occurs at lower temperatures compared with the conventional sintering method. No mechanical tests of the samples were made, but the samples were compact enough to cut thin plates of the magnetocaloric material without breaking them. 

**Microwave-assisted sol-gel Pechini.** Stoichiometric quantities of lanthanum nitrate (La(NO_3_)_3.6_H_2_O) 99.999%, calcium nitrate (Ca(NO_2_)_3.4_H_2_O) 99.0%, strontium nitrate (Sr(NO_2_)_3_) 99.0%, and manganese nitrate (Mn(NO_2_)_3.4_H_2_O) 97.0% were dissolved in distilled water with continuous magnetic stirring; after 15 min, citric acid and ethylene glycol were added and stirred for 30 min. The solution was transferred to vials of an Anton Paar Multiwave PRO microwave system (Anton Paar, Graz, Austria), and the controlled-temperature reaction option was set at 250 °C for 1 hour. Subsequently, the reaction product was heated with a heating rate of 2 °C/min and maintained at 110 °C for 1 h; then, it was heated with a heating rate of 30 °C/min and maintained at 800 °C for 3 h. Finally, the powders were milled, pressed, and sintered at 900 °C/3h, 1000 °C/3h, 1100 °C/3h, and 1200 °C/3h [25].

**Solid-state reaction route (with planetary mill).** Nominal compositions were prepared from the following high-purity oxide and carbonate powders: La_2_O_3_ (99.99%), Mn_2_O_3_ (99%), SrCO_3_ (99.9%), and CaCO_3_ (99.99%). These materials were mixed in an agate mortar and then were calcined in a two-step heat treatment process: 15 h at 750 °C and 18 h at 950 °C, respectively, with intermediate milling between heat treatments in a planetary ball mill Fritsh PULVERISETTE 7 (Fritsch, Idar-Oberstein, Germany). Finally, the powders were pressed and sintered at 1300 °C for 24 h [26].

**Solid-state reaction route (with attritor mill).** Nominal compositions were prepared from the following high-purity oxide and carbonate powders: La_2_O_3_ (99.99%), Mn_2_O_3_ (99%), SrCO_3_ (99.9%), and CaCO_3_ (99.99%). These materials were combined and milled in isopropanol for 1 h at 300 RPM in an attrition mill model HDDM-01 from Union Process. After drying, the powders were calcined at 800 °C for 18 h. A second milling was carried out with the same conditions as the first milling. After drying, the powders were sintered at 1100 °C for 24 h.

Room-temperature X-ray diffraction patterns were measured in a PANalytical diffractometer, model X´Pert PRO (PANalytical, Almelo, Netherlands), with Cu-Kα radiation (λ = 1.5406 A°) in an angular interval from 20° to 100° and with an angular step of 0.02° and a time of 15 s per step. Surface morphology and particle sizes of samples were studied by field-emission scanning electron microscopy, JEOL model JSM-7401F (JEOL, Tokio, Japan). Magnetic measurements were carried out in a Quantum Design Physical Properties Measurement System (PPMS) (Quantum Design, San Diego, CA, USA). Thermomagnetic curves were measured with a constant magnetic field of 70 Oe, in the temperature range of 200–350 K with a heating rate of 1 K/min. Isothermal magnetization curves were measured in the temperature range of 250–340 K, with applied magnetic fields up to 5 T. All the characterizations were carried out under the same conditions. A MATLAB program (MathWorks, Natick, MA, USA) was developed to calculate magnetic entropy changes and Arrot plots from measured isothermal magnetization curves.

## 3. Results and Discussion

**Figure 1:**
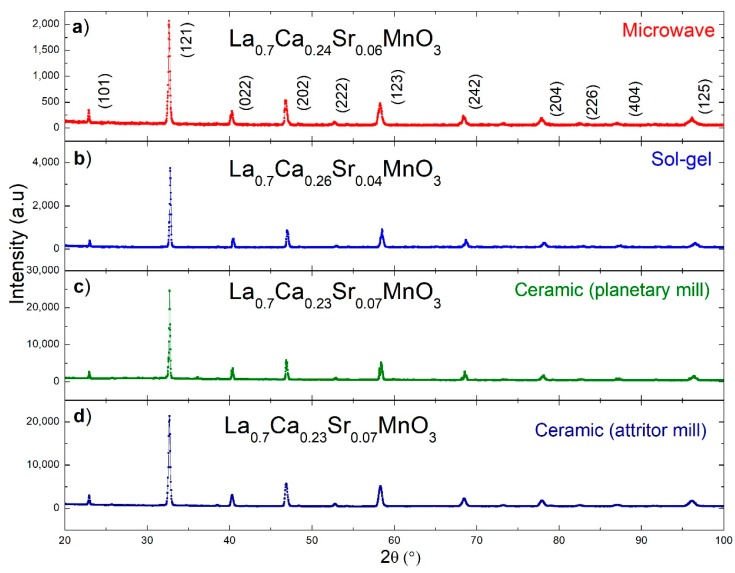
Powder X-ray diffraction patterns of the manganites obtained by different methods: (**a**) microwave-assisted sol-gel Pechini method; (**b**) sol-gel Pechini chemical synthesis; (**c**) solid-state reaction with planetary mill; and (**d**) solid-state reaction with attritor mill.

Figure 1 shows the powder X-ray diffraction patterns for manganites, La_0.7_Ca_0.3−x_Sr_x_MnO, obtained by different manufacturing routes (1a, 1b, 1c, and 1d). The diffraction peaks were indexed to the crystallographic data sheet PDF: 01-071-5292, corresponding to the orthorhombic system with spatial group Pnma. In all cases, pure manganite phase was obtained.

**Figure 2:**
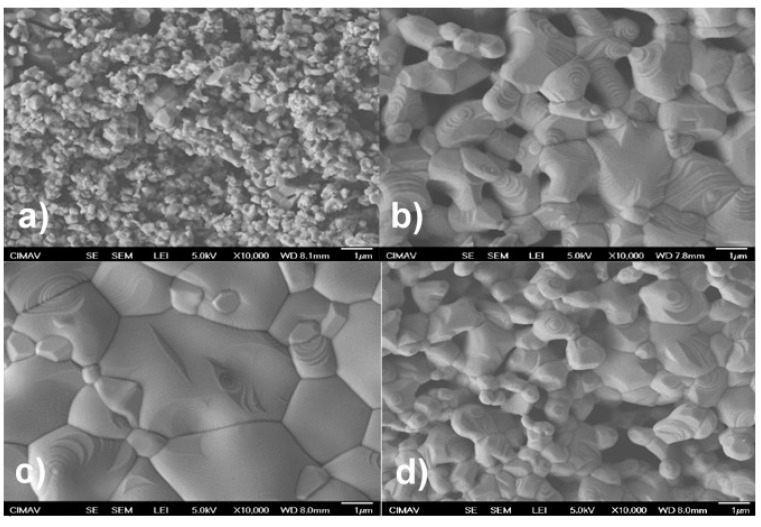
SEM images of manganites manufactured by: (**a**) microwave-assisted sol-gel; (**b**) Pechini sol-gel; (**c**) solid-state reaction with planetary mill; and (**d**) solid-state reaction with attrition mill.

**Figure 3:**
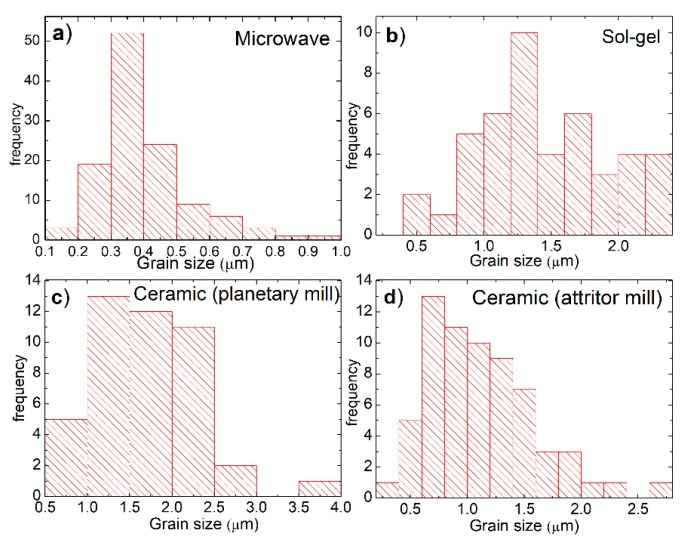
Particle average size histograms from the observed SEM images of manganites manufactured by: (**a**) microwave-assisted sol-gel; (**b**) Pechini sol-gel; (**c**) solid-state reaction with planetary mill; and (**d**) solid-state reaction with attrition mill.

Figure 2 shows the surface morphology observed by scanning electron microscopy for manganites manufactured by microwave-assisted sol-gel Pechini method (Figure 2a), sol-gel Pechini chemical synthesis (Figure 2b), solid-state reaction with planetary mill (Figure 2c), and solid-state reaction with attritor mill (Figure 2d). A quantitative particle distribution histogram for the different fabrication routes is shown in Figure 3.

Figure 2a shows particles with irregular shapes and some facets, as well as agglomerated particles. The average crystallite size, as determined by the Debye-Scherrer method from the X-ray diffraction pattern, was 26 nm, while the average particle size, determined by SEM, was 0.4 µm. 

Figure 2b,d shows a granular morphology with an average particle size of 1.44 μm and 1.12 μm for manganite manufactured by Pechini sol-gel and by the solid-state reaction with attrition mill, respectively. Figure 2c shows a granular morphology limited by grain boundaries, with an average particle size of 1.68 μm. Figure 2b–d also shows, on the surface of some grains, terraces in the form of parallel and equidistant bands, possibly formed by a process of evaporation and condensation (as is the case when crystals grow from the vapor phase) [27,28]. Manganite manufactured by chemical methods have a narrower particle-size distribution, while manganite manufactured by solid-state reaction methods have a wider particle-size distribution, as noted in Figure 3.

A summary of the average grain size of the particles obtained by all fabrication routes is provided in Table 1. 

**Figure 4:**
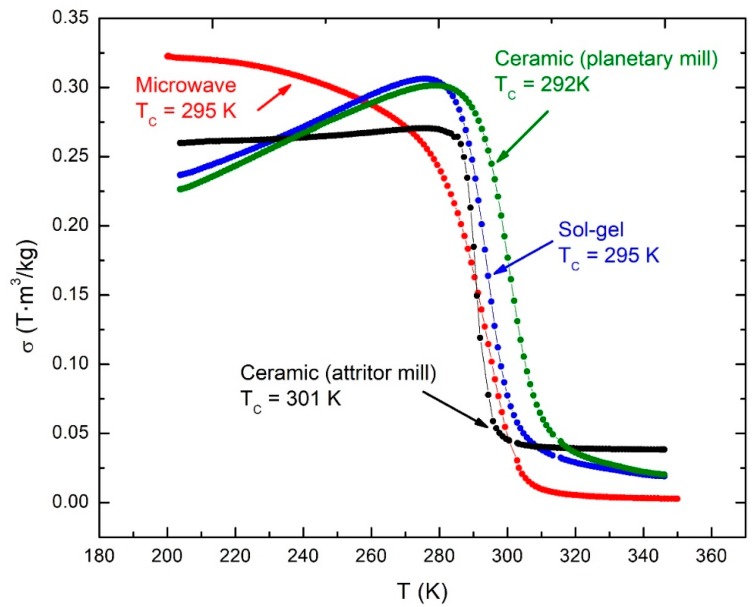
Thermomagnetic curves of lanthanum-calcium-strontium manganites, manufactured by different routes. These curves were measured with an applied magnetic field of 7.0 mT.

Figure 4 shows manganite thermomagnetic curves for samples manufactured by microwaves-assisted sol-gel, Pechini sol-gel, solid-state reaction with planetary mill, and solid-state reaction with attrition mill. Curie temperatures were determined from the inflection point of the curves by means of the dσ/dT derivative. 

As shown, the thermomagnetic curves of manganites manufactured by Pechini sol-gel and solid-state reactions using planetary or attrition mill show an anomaly in their behavior, since with increasing temperature the magnetization first increases and then it begins to decrease, when the magnetization should always decrease with increasing temperature. This phenomenon is related to magnetic fields trapped in the PPMS superconducting coil. This problem appears when the sample is first cooled without an applied magnetic field. In fact, a certain magnetic field may be trapped in the superconducting coil in the opposite direction to the magnetic field applied when the measurement is made on heating. This trapped magnetic field aligns the sample magnetization during cooling in the opposite direction to the magnetic field of 7.0 mT, which is applied to measure. 

Since magnetic anisotropy and coercivity of ferromagnetic materials increase as temperature decreases, it turns out that the constant magnetic field of 7.0 mT, applied at the lowest temperature, is not sufficiently intense enough to align the magnetization at the very moment when it is applied, and only after sufficient magnetic anisotropy and coercivity decrease with an increase in temperature can this small field finally align the magnetization. This small magnetic field trapped by the superconducting coil can be eliminated with a special low magnetic field option, but our system does not have this option. Sometimes, this small magnetic field trapped by the superconducting coil can be eliminated by performing a procedure of alternating cycles of decreasing magnetic field amplitude. Also, if we apply a magnetic field greater than 7.0 mT to measure the thermomagnetic curves, the relatively large low-temperature anisotropy field and coercivity can be overcome, thus avoiding this anomaly, but in that case the initial magnetic state of the sample will be altered. However, this phenomenon occurs at low temperatures and does not overlap with the rapid magnetization change near the Curie temperature, which is what we are interested in. This anomaly is not observed for the manganites manufactured by the microwave-assisted sol-gel, which produces nanometric-sized crystallites, as determined by the X-ray diffraction Scherrer method; we suppose, therefore, that in this case, the superparamagnetic behavior suppresses the effect of the high values of anisotropy and coercivity at lower temperatures.

The magnetocaloric effect in the different manufactured manganites was determined as the magnetic entropy changed under isothermal conditions using the measured isothermal magnetization curves as a function of the applied magnetic field for a discrete set of temperatures. A MATLAB program was developed to numerically calculate the magnetic entropy change as a function of temperature for values of magnetic field change of 1, 2, 3, 4, and 5 T. The program also calculates the Arrot plots. The MATLAB program uses the equation:

(1) $$\Delta S_{M}=\int_{H_{1}}^{H_{2}}\left(\frac{\partial M}{\partial T}\right)\mathrm{dH}$$

This is the thermodynamic equation for the magnetic entropy change ∆S_M_, which is strictly valid only for reversible thermodynamic processes. This equation does not take into account irreversible processes associated with magnetic hysteresis. Irreversible processes are more pronounced for first-order magneto-structural transitions than for the second-order magnetic transitions that occur in these manganites.

**Figure 5:**
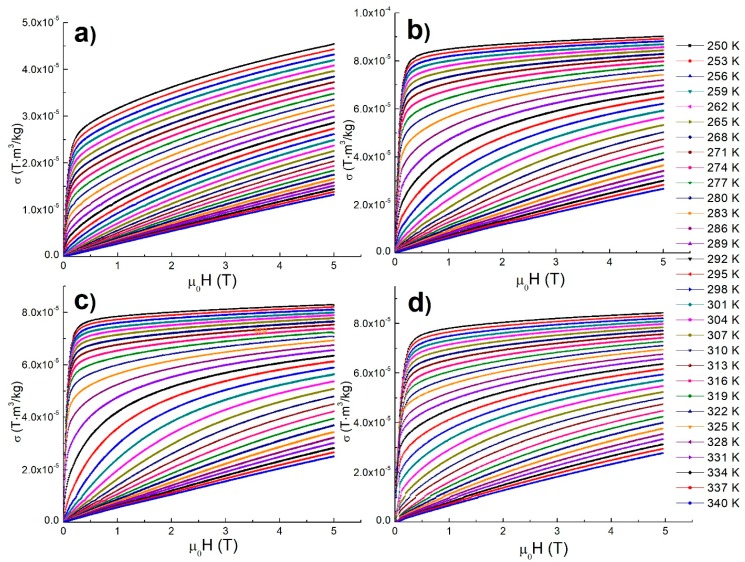
Magnetization versus applied magnetic field, measured at different temperatures, for manganites by (**a**) microwave-assisted sol-gel, (**b**) Pechini sol-gel, (**c**) solid-state reaction with planetary mill, and (**d**) solid-state reaction with attrition mill.

Figure 5 shows isothermal magnetization curves as a function of the applied magnetic field up to 5 T, in the temperature range from 250 K to 340 K and with a 3 K step for La-Ca-Sr manganites manufactured by microwave-assisted sol-gel, Pechini sol-gel, solid-state reaction with planetary milling, and solid-state reaction with attrition milling. 

In the isothermal magnetization curves below the Curie temperature, a ferromagnetic behavior is clearly observed, where the magnetization increases rapidly with relatively small applied magnetic fields, and subsequently, the magnetization tends to saturate for higher applied magnetic fields. However, even for the maximum applied magnetic field of 5 T, the samples still do not completely reach saturation, and this effect is more noticeable for manganites manufactured by the microwave-assisted sol-gel method due to the nanometric size of its crystals that creates a surface anisotropy in the particles. On the other hand, for isothermal magnetization curves above the Curie temperature, a linear paramagnetic behavior with the applied magnetic field is observed. Around the Curie temperature, the isothermal magnetization curves are more separated due to the more rapid variation of magnetization with temperature in this region.

**Figure 6:**
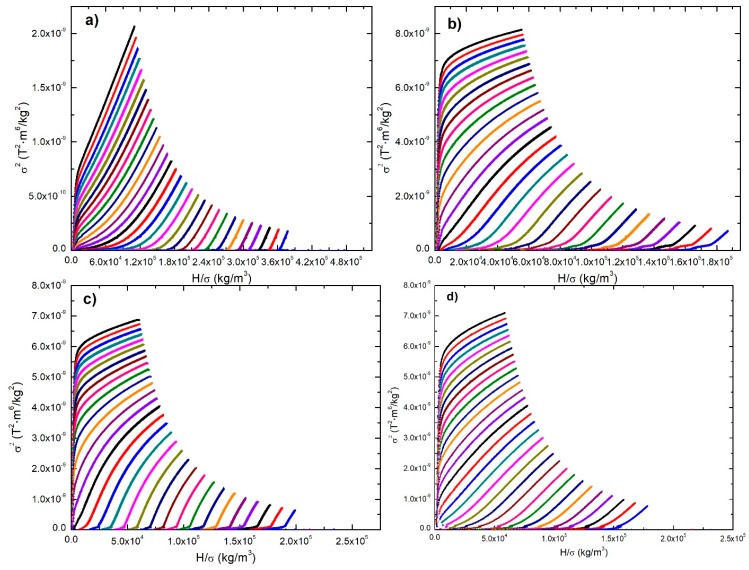
Arrot curves plots (σ^2^ vs H/σ) for manganites manufactured by (**a**) microwave-assisted sol-gel, (**b**) Pechini sol-gel, (**c**) solid-state reaction with planetary mill, and (**d**) solid-state reaction with attritor mill.

Figure 6 shows Arrot plots. Arrot plots are plots of σ^2^ vs H/σ, where σ is specific magnetization in (T^2^⋅m^6^/kg^2^) and H is the magnetic field intensity in (kg/m^3^). The Curie temperature can be determined using the Arrot curve that passes through the origin of the Arrot plot. In this way, Curie temperatures of 280 K, 292 K, 292 K, and 301 K were determined for manganite manufactured by microwave-assisted sol-gel, Pechini sol-gel, solid-state reaction with planetary mill, and solid-state reaction with attritor mill, respectively. In addition, the combined use of Arrot plots and the Banerjee criterion allow to determine the order of the transition. According to the Banerjee criterion, the magnetic transition is of second order if the slopes of the Arrot curves are positive, while the magnetic transition is of first order if the slopes of the Arrot curves are negative. From the Arrot plot in Figure 6 it can be observed that in all cases, the magnetic transition is second-order, since the curve slopes are all positive.

**Figure 7:**
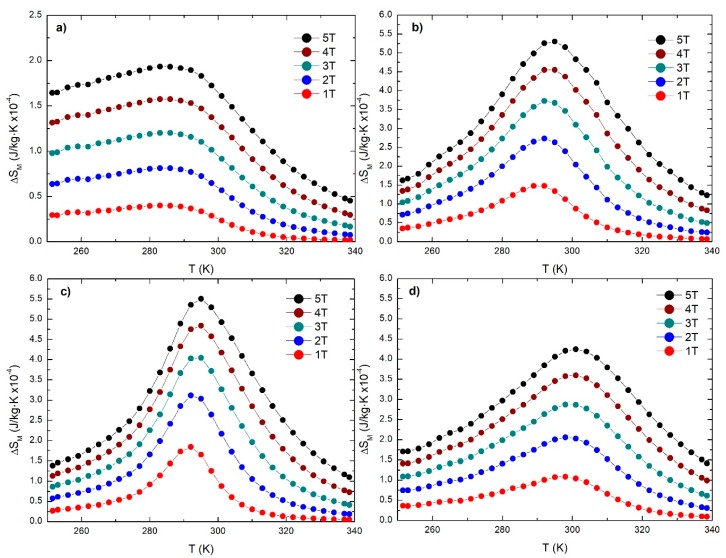
∆S_M_ (T) curves with applied field of 10 kOe to 50 kOe for (**a**) microwaves-assisted sol-gel, (**b**) Pechini sol-gel, (**c**) solid-state reaction with planetary mill and (**d**) solid-state reaction with attrition mill.

Figure 7 shows the magnetic entropy change curves for manganites manufactured by a microwave-assisted sol-gel, Pechini sol-gel, solid-state reaction with planetary mill, and solid-state reaction with attrition mill. It can be observed that the maximum magnetic entropy changes occur at temperatures of 286 K, 295 K, 295 K, and 301K for manganite manufactured by microwave-assisted sol-gel, Pechini sol-gel, solid-state reaction with planetary mill, and solid-state reaction with attrition mill, respectively, which are near to the Curie temperatures determined from the thermomagnetic curves in Figure 3. The (∆S_M_)_max_ value increases as the applied magnetic field increases. The (∆S_M_)_max_ values are 1.9, 5.3, 5.5, and 4.2 × 10^−4^ J/kg·K for manganite manufactured by microwave-assisted sol-gel, Pechini sol-gel, solid-state reaction with planetary mill, and solid-state reaction with attrition mil, respectively, for a magnetic field change of 5 T. It can be observed that the higher value corresponds to manganite manufactured by a solid-state reaction with planetary mill.

Fullwidth of the ΔSM peak is determined as the peak width measured at half the peak height (FWHM). Observe the particular behavior of the thermomagnetic curve (see Figure 3) and of the ∆S_M_(T) curves (see Figure 6) for the case of manganites manufactured by microwave-assisted sol-gel. In this case, the fall of magnetization in the thermomagnetic curve extends widely in temperature and (∆S_M_)_max_ are not well-defined, especially on the low-temperature side. This particular behavior is related to the appearance of a dependence of Curie temperature with crystallite size when the crystallite size is of the order of tens of nanometers. This means that if the system is not well-monodispersed, then there will be a Curie temperature distribution and a consequent smoothing of the maxima.

**Figure 8:**
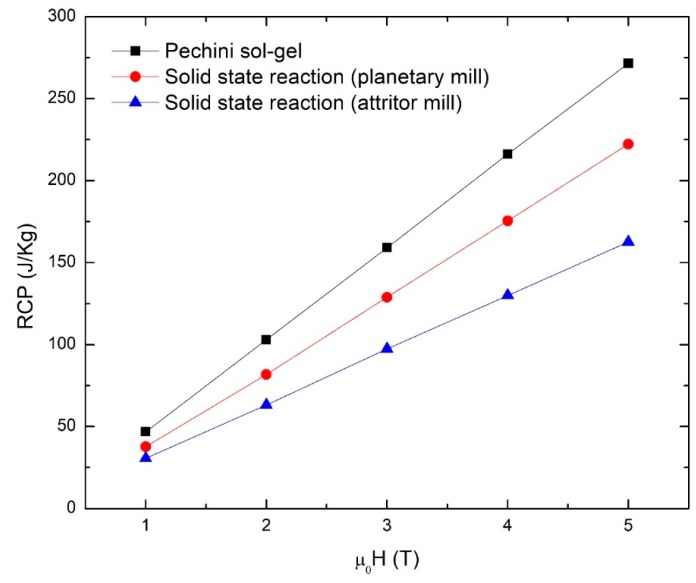
Relative cooling power (RCP) as a function of the magnetic field change of manganites manufactured by Pechini sol-gel, solid-state reaction with planetary mill, and solid-state reaction with attrition mill.

Figure 8 shows the Relative Cooling Power (RCP) as a function of the applied magnetic field. The RCP is defined as the product of (∆S_M_)_max_ by ($\delta T_{\mathrm{FWHM}}$), where ($\delta T_{\mathrm{FWHM}}$) is the temperature range corresponding to the FWHM [29].

(2) $$\mathrm{RCP}={\left(\Delta S_{M}\right)}_{\max}{\delta T}_{\mathrm{FWHM}}$$

The RCP is a measure of the total cooling energy of the magnetocaloric material given in (J/kg). As can be seen in Figure 7, the highest RCP values correspond to the manganite manufactured by the Pechini sol-gel, followed by the manganite manufactured by the solid-state reaction with a planetary mill and then by the manganite manufactured by a solid-state reaction with an attrition mill. It was not possible to determine the RCP of the manganite manufactured by microwave-assisted sol-gel because the fullwidth could not be determined at half (∆S_M_)_max_.

The RCP values depend on the chemical composition, size, and size distribution of manganite particles. Size and size distribution depend on the manufacturing method. Although manganites manufactured by a solid-state reaction with an attrition mill have a similar particle size to the manganites manufactured by the Pechini sol-gel, and their ΔS_M_ curves show similar fullwidth values, a lower (ΔS_M_)_max_ value—and therefore, lower RCP—is observed.

**Figure 9:**
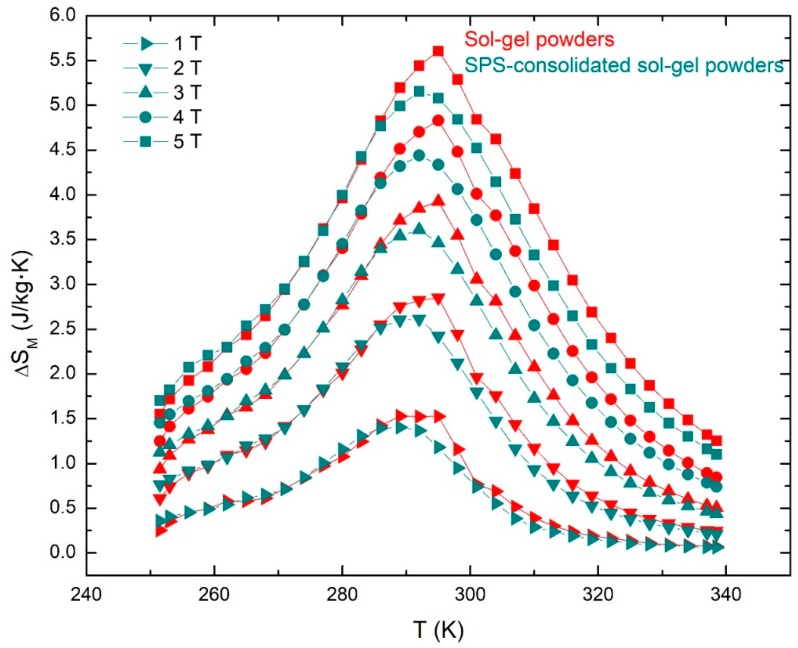
∆S_M_ (T) curves with applied field of 10 kOe to 50 kOe for sol-gel powders and Spark Plasma Sintering (SPS)-consolidated sol-gel powders.

Figure 9 compares the magnetic entropy changes of powders and SPS-consolidated tablets of manganites manufactured by the Pechini sol-gel method. 

(ΔS_M_)_max_ values of 5.3 × 10^−4^ J/kg·K and 5.16 × 10^−4^ J/kg·K were obtained for powders and SPS-consolidated tablets, respectively, for a magnetic field change of 5 T. These magnetic entropy changes values are not very different, indicating that the microstructure was not significantly affected by the SPS process. Even more important, very good mechanical properties were obtained by SPS consolidation, allowing the manufacture of manganite plates for the magnetic regenerator by cutting with a diamond disk.

## 4. Conclusions

La(Ca,Sr)MnO manganites, prepared by four different methods, all belong to the orthorhombic crystal system, and in all cases, pure manganite phase was obtained. From SEM micrographs, manganite obtained by chemical methods present a narrower particle-size distribution than manganite obtained by solid-state reaction methods. Curie temperature for manganite manufactured by microwave-assisted sol-gel, Pechini sol-gel, and solid-state reaction with planetary mill was ~295 K, while for a solid-state reaction with an attritor it was 301 K; thus, there was found to be no correlation between the particle size and the Curie temperature. The microwave-assisted sol-gel manganite has a magnetization lower than the other manganites; however, no sample reached the maximum saturation value. All samples gave a positive slope in the Arrot plots, corresponding to a second-order FM-PM phase transition. The values of (∆S_M_)_max_, for a magnetic field change of 5 T, are 1.9, 5.3, 5.5, and 4.2 × 10^−4^ J/kg·K for manganite manufactured by microwave-assisted sol-gel, Pechini sol-gel, solid-state reaction with planetary mill, and solid-state reaction with attrition mill, respectively. The Pechini sol-gel manganite presents higher values of RCP than the solid-state reaction manganite, because its entropy change curves are smaller, but wider, associated to the particle size obtained by the preparation method. The best results of magnetic and magnetocaloric properties were obtained for samples synthesized by the Pechini sol-gel and the solid-state reaction (solid-state reaction with planetary mill) methods. The SPS technique showed that it was able to produce consolidated solids for applications in active magnetic regenerative refrigeration easier and faster compared with other compaction methods.

## Figures and Tables

**Table 1:** Average grain size of the manganites prepared by the different methods.

Preparation Method	Average Grain Size (μm)	Standard Deviation (μm)
Microwave	0.40	0.13
Sol-gel	1.44	0.47
Ceramic (planetary mill)	1.68	0.60
Ceramic (attritor mill)	1.12	0.47
